# First-trimester metabolic profiling of gestational diabetes mellitus: insights into early-onset and late-onset cases compared with healthy controls

**DOI:** 10.3389/fmolb.2024.1452312

**Published:** 2025-01-15

**Authors:** Danuta Dudzik, Vangeliya Atanasova, Coral Barbas, Jose Luis Bartha

**Affiliations:** ^1^ Department of Biopharmaceutics and Pharmacodynamics, Faculty of Pharmacy, Medical University of Gdańsk, Gdańsk, Poland; ^2^ Division of Maternal and Fetal Medicine, Fundación Para la Investigación Biomédica, La Paz University Hospital, Madrid, Spain; ^3^ Department of Chemistry and Biochemistry, Centre for Metabolomics and Bioanalysis (CEMBIO), Facultad de Farmacia, Universidad San Pablo-CEU, CEU Universities, Urbanización Montepríncipe, Madrid, Spain

**Keywords:** gestational diabetes mellitus, pregnancy complications, biomarkers, metabolomics, metabolism, mass spectrometry, metabolic phenotyping, diabetes prediction

## Abstract

**Introduction:**

Gestational diabetes mellitus (GDM) is a global health concern with significant short and long-term complications for both mother and baby. Early prediction of GDM, particularly late-onset, is crucial for implementing timely interventions to mitigate adverse outcomes. In this study, we conducted a comprehensive metabolomic analysis to explore potential biomarkers for early GDM prediction.

**Methods:**

Plasma samples were collected during the first trimester from 60 women: 20 with early-onset GDM, 20 with late-onset GDM, and 20 with normal glucose tolerance. Using advanced analytical techniques, including liquid chromatography-tandem mass spectrometry (LC-MS/MS) and gas chromatography-mass spectrometry (GC-MS), we profiled over 150 lipid species and central carbon metabolism intermediates.

**Results:**

Significant metabolic alterations were observed in both early- and late-onset GDM groups compared to healthy controls, with a specific focus on glycerolipids, fatty acids, and glucose metabolism. Key findings revealed a 4.0-fold increase in TG(44:0), TG(46:0), TG(46:1) with *p*-values <0.001 and TG(46:2) with 4.7-fold increase and *p*-value <0.0001 as well as changes in several phospholipids as PC(38:3), PC(40:4) with 1.4-fold increase, *p* < 0.001 and PE(34:1), PE(34:2) and PE(36:2) with 1.5-fold change, *p* < 0.001 in late-onset GDM.

**Discussion:**

Observed lipid changes highlight disruptions in energy metabolism and inflammatory pathways. It is suggested that lipid profiles with distinct fatty acid chain lengths and degrees of unsaturation can serve as early biomarkers of GDM risk. These findings underline the importance of integrating metabolomic insights with clinical data to develop predictive models for GDM. Such models could enable early risk stratification, allowing for timely dietary, lifestyle, or medical interventions aimed at optimizing glucose regulation and preventing complications such as preeclampsia, macrosomia, and neonatal metabolic disorders. By focusing on metabolic disruptions evident in the first trimester, this approach addresses a critical window for improving maternal and fetal outcomes. Our study demonstrates the value of metabolomics in understanding the metabolic perturbations associated with GDM. Future research is needed to validate these biomarkers in larger cohorts and assess their integration into clinical workflows for personalized pregnancy care.

## Introduction

Gestational diabetes mellitus (GDM) defined as hyperglycemia first recognised in pregnancy (World Health Organization, 1999) is a complex condition and a growing health issue worldwide. This definition includes undiagnosed pre-pregnancy hyperglycemia and glucose intolerance with first onset during pregnancy, being one of the most commonly diagnosed pregnancy complications. According to a recent report by the International Diabetes Federation (IDF) Atlas, gestational diabetes mellitus affects 2% to as much as 40% of pregnancies worldwide, depending on the diagnostic and screening criteria, which vary and remain controversial. These discrepancies complicate the comparison and interpretation of research findings (IDF Diabetes Atlas, 2021). Recently, there has been a shift towards adopting the International Association of Diabetes and Pregnancy Study Groups (IADPSG) criteria (IDF Diabetes Atlas, 2021), which has led to an increase in the reported incidence of GDM (IDF Diabetes Atlas, 2021; Saeedi et al., 2021). GDM contributes to several short- and long-term health consequences both for the mother (e.g., cesarean section, preeclampsia, metabolic syndrome, cardiovascular disease) (Damm, 2009; Song et al., 2018; Kramer et al., 2019; Yang and Wu, 2022) and baby (e.g., macrosomia, neonatal hypoglycemia, metabolic syndrome, cardiovascular disease, diabetes) (Bianco and Josefson, 2019; Meek, 2023; Rodolaki et al., 2023; American Diabetes Association Professional Practice Committee, 2024). Therefore, identifying any glucose impairment, especially in early pregnancy, can improve clinical outcomes (Sweeting et al., 2022). It has been demonstrated that patients with gestational diabetes discovered in early pregnancy represent a higher-risk subgroup in terms of associated pregnancy complications (Bartha et al., 2000; Bartha et al., 2003). Bartha et al. were among the first to suggest the hypothesis that this group is mainly represented by type 2 pregestational diabetes and to a lesser extent actual pregnancy-induced glucose intolerance (Bartha et al., 2003). Key epidemiological factors, including rising obesity rates, advanced maternal rage and increased instances of undiagnosed pre-pregnancy diabetes, identified during pregnancy are critical for identifying women at risk for GDM (American Diabetes Association Professional Practice Committee, 2024). With the global GDM concern, it becomes crucial to raise the efforts for early detection of glucose intolerance in pregnancy through studies focusing on the development of new predictive models for improved and accurate GDM diagnosis.

In such a premise, metabolomics offers excellent research solutions for elucidating biochemical changes in human health and disease. The global untargeted metabolomics complements information derived from genomics, transcriptomics and proteomics, supporting a system “omics” approach that might impact our ability to understand pathological conditions including pregnancy complications. Remarkable, disease-specific metabolic signatures can be captured with the potential to drive new developments in clinical biomarkers.

**FIGURE 1:**
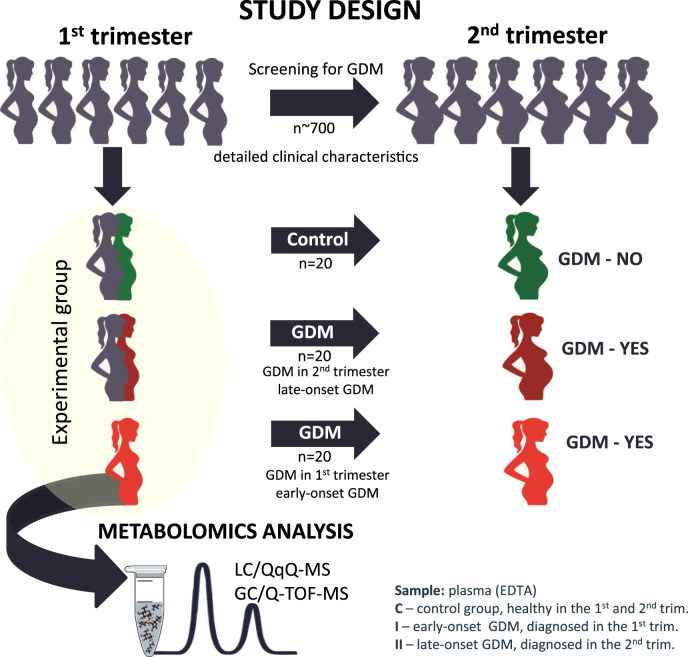
The graphic representation of the study design from the selection of the studied cohort to the metabolomics analysis.

Our study aimed to look for differences at the metabolome level in the first trimester of pregnancy to identify metabolic alterations that could indicate impaired glucose tolerance associated with early and late-onset GDM. Our clinical and research interests focus on giving new insights and directions for the future development of novel prognostic strategies for improved GDM recognition. The graphic representation of the study design is illustrated in Figure 1.

## Materials and methods

### Study population

This is a longitudinal prospective cohort study from the Obstetrics and Gynecology department, La Paz University Hospital in Madrid, carried out between December 2017 and June 2020. The protocol of the study was approved by the local Ethics and Research Committee from La Paz University Hospital in Madrid, Spain. All eligible pregnant women without known diabetes, who were older than 16 years of age and signed the informed consent in their first trimester were invited to participate in the study. Exclusion criteria were maternal age under 16 years, gestational age >14 weeks, multiple pregnancies, known foetal defect at the time of recruitment and pre-gestational diagnosis of Diabetes Mellitus. Women with other medical co-morbidities were excluded from the study. The recruitment took place in the Obstetrical clinics of La Paz University Hospital and the control of the GDM pregnancies was carried out in the Diabetes in Pregnancy unit. The healthy control group had pregnancies followed up in Routine Obstetrical clinics. For each participant enrolled a record of maternal and gestational age at the time of recruitment, maternal characteristics including height, weight, body mass index (BMI), family history of diabetes, obstetrical record including previous history of GDM, past medical history, glycemic test results and type of GDM management including insulin or diet treatment, the course of gestation (presence or absence of further pregnancy complications), way of delivery, gestational age at birth, newborn`s weight, Apgar score and umbilical artery pH at birth was taken. The diagnosis of gestational diabetes mellitus was made based on a two-step approach: screening by 1-h 50 g glucose challenge test (GCT) and diagnostic 3-h oral glucose tolerance test (OGTT) with 100 g glucose load for the GCT positive (glycaemia >140 mg/dL). The diagnostic criteria of Carpenter and Coustan (1982) with two or more glucose plasma levels higher than fasting glucose of 95 mg/dL, 1-h of 180 mg/dL, 2-h of 155 mg/dL, and 3-h glycaemia of 140 mg/dL were applied for this study.

La Paz University Hospital has a strategy of first-trimester screening for gestational diabetes mellitus in high-risk patients. The high-risk group for GDM is determined by the presence of any of the following risk factors: maternal age older than 35 years; maternal pre-pregnancy BMI >30 kg/m2; GDM in previous pregnancy; newborn from previous pregnancy with birth weight >4,500 g; first-grade family history of diabetes mellitus. The high-risk patients in this study were screened with CGT (O`Sullivan test) in their first trimester according to the local protocol. The screen-positive patients had a 3-h OGTT with 100 g glucose. The patients with positive results according to CC criteria were included as early-onset or first-trimester GDM. Patients who screened positive but who had a negative diagnostic test in the early group had a 3-h OGTT at 24–28 weeks instead of GCT. Thus the overall prevalence of GDM in the cohort of patients was 5%.

More than 700 women matched with the inclusion criteria were examined and the control group of healthy pregnant normal glucose tolerance women (C, n = 20), early-onset first-trimester GDM (I, n = 20) and late-onset, first-trimester asymptomatic women that developed GDM in second-trimester (II, n = 20) were selected to perform metabolomics analysis. The groups were initially matched for age, pre-gestational BMI and parity. Venous fasting blood was drawn from each eligible patient in the first trimester (≤14 weeks’ gestation) into EDTA-containing tubes. Samples were stored at −80°C until analysis.

### Chemicals and reagents

Methanol (MeOH) MS grade was obtained from Sigma Aldrich (Steinheim, Germany). Acetonitrile (ACN) MS grade (Fluka Chromassol, Spain), pyridine (Carlo Erba Reagents SAS, France), 2-propanol (PrOH) (Fischer, Austria), ammonia (NH_3_, 28%) and glacial acetic acid (AcAc) were supplied by VWR Chemicals (Pennsylvania, United States). Ethyl acetate (EtAc) and formic acid (FA, 99.8%) were obtained from Honeywell (New Jersey, United States). Heptane MS grade, C18:0 Methyl stearate, N, O-Bis (trimethylsilyl) trifluoroacetamide (BSTFA) with 1% trimethylchlorosilane (TMCS) obtained from Sigma Aldrich. A mixture of alkanes standards (Supelco, United States), a mixture of methyl acids and fatty acids (FAME C8–C22), O-methoxamine, 4-nitrobenzoic acid and tricosane were obtained from Sigma Aldrich. Ultra-pure water was generated with a Milli-Q Plus 185 water purification system (Millipore S.A., Molsheim, France)). SPLASH® Lipidomix® Mass Spec Standard, an internal standard mixture containing 18:1 (d7) LPE, 15:0–18:1 (d7) PC, 15:0–18:1 (d7) PE, 15:0–18:1 (d7)-15:0 TG, 18:1 (d7) Chol Ester, 18:1 (d7) LPC, 18:1 (d9) SM, 18:1 (d7) DG, 15:0–18:1 (d7)-PA, 15:0–18:1 (d7)-PG, 15:0–18:1 (d7)-PI, 15:0–18:1 (d7)-PS, 18:1 (d7)-MG and cholesterol (d7) was obtained from Avanti® Polar Lipids, Inc. (Alabama, United States), and was 20 times diluted (25 µL/500 µL) in MeOH before analysis. The standard working solution was stored at −20°C.

### Sample preparation

The plasma samples were randomized, thawed on ice and thoroughly vortex-mixed. For lipid extraction 10 µL of the internal standard mixture (SPLASH® Lipidomix® Mass Spec Standard) was added to 10 µL of each plasma sample. Protein precipitation and lipid extraction were performed with 800 µL solvent mixture (EtAc:EtOH, 2:1), followed by centrifugation (13,700 rpm, 10 min, 15°C). 250 μL of the supernatant was transferred to chromatographic vials for LC-MS/MS analysis (Konjevod et al., 2022).

Samples for GC-MS analysis were prepared as previously described (Rey-Stolle et al., 2021). Briefly, proteins were precipitated by mixing 1 volume of plasma with 3 volumes of cold acetonitrile containing 4-nitrobenzoic acid (IS) (1:3), followed by methoximation with O-methoxyamine hydrochloride (15 mg/mL) in pyridine, and silylation with N,O bis(trimethylsilyl)trifluoroacetamide (BSTFA) with 1% trimethylchlorosilane (TMCS). 100 μL of heptane containing 20 ppm of tricosane (IS) was added to each vial and vortex-mixed before GC-MS analysis.

Blank and QC samples were prepared for each analytical platform following specified quality assurance criteria (Dudzik et al., 2018). A pool of plasma (QC) was prepared by mixing an equal volume of all experimental samples, following the same metabolite extraction protocols. All experimental samples were randomized and QCs were injected at the beginning, every 5 samples, and at the end of the batch. The blank samples were analyzed at the beginning and the end of each analytical run.

### LC-MS/MS analysis

The lipidomics analysis was performed on a 1260 Infinity high-pressure liquid chromatography system equipped with a degasser, a binary pump, and an autosampler, interfaced to a 6470 triple-quadrupole mass spectrometer (Agilent Technologies, CA, United States) (Konjevod et al., 2022). 5 μL of the extracted plasma samples were injected into a Gemini® C6-phenyl column (3.5 µm, 2.1 mm × 15 cm, Phenomenex®), maintained at 60°C. The mobile phase consisting of 1 mmol/L ammonium acetate (NH_4_Ac) in 30:70 MeOH:H_2_O (phase A) and MeOH (phase B), both containing 0.1% formic acid (*v/v*), with a flow rate of 0.6 mL/min and the gradient started at 0% B, increasing to 100% B in 1 min, then held until 12 min. Starting conditions were reached at 13 min, and 5 min of re-equilibration was applied. The electrospray ionization (ESI) was operated in positive ion mode and the following parameters: gas temperature: 250°C, gas flow rate: 7 L/min, nebulizer pressure: 30 psi, sheath gas temperature: 350°C, sheath gas flow rate: 12 L/min, capillary voltage: 4000 V, and nozzle voltage: 500 V. MS/MS data were acquired in dynamic multiple reaction monitoring modes (dMRM) by using the most abundant precursor and product ions of each compound. The list of 156 targeted compounds with the acquisition parameters is presented in Supplementary Table S1.

### GC/Q-TOF-MS analysis

The analysis was performed with a GC system (7890B, Agilent Technologies) coupled to an accurate mass Q-TOF mass spectrometer (7250, Agilent Technologies). The derivatized samples (1 μL) were injected (autosampler 7693, Agilent Technologies) in split mode (ratio 1:12) into a deactivated glass-wool split liner (Restek 20782) in a GC column DB5-MS (30 m length, 0.25 mm internal diameter, 0.25 μm film 95% dimethyl/5% diphenylpolysiloxane) coupled to a pre-column (10 mJ & W integrated with Agilent 122-5532G). The injector port was held at 250°C, and the helium carrier gas flow rate was set at 0.917 mL/min. The temperature gradient was programmed as follows: the initial oven temperature was set to 60°C (held for 1 min), with a ramping rate of 10°C/min up to 325°C. The system was allowed to cool down for 10 min before the next injection. The total analysis time was 37.5 min per sample. The detector transfer line, the filament source and the quadrupole temperature were set to 280, 200°C and 150°C, respectively. MS detection was performed in electron impact (EI) mode at −70 eV. The mass spectrometer was operated in scan mode over a mass range of 40–600 *m/z* at a rate of 10 scan/s (Rey-Stolle et al., 2021).

Fatty Acid Methyl Esters (FAME) mix was analyzed at the beginning of the analytical batch. This procedure was performed to establish retention index markers across chromatograms, ensuring accurate alignment and identification of metabolites using Fiehn’s library.

### Data processing

The acquired LC-MS data were reprocessed with the MassHunter Qualitative Analysis and MassHunter Quantitative Analysis software (Ver. B10.00, Agilent Technologies). The MRM signal runs as well as pressure curves were visually inspected to confirm homogeneity and reproducibility across the chromatograms. In the next step peaks of the targeted compounds were integrated to determine the peak area size for each analyzed metabolite. The representative MRM chromatogram is presented in Supplementary Figure S1.

Spectral deconvolution (GC-MS) and annotation of metabolites comparing the mass spectrum obtained with those of a compound library (Fiehn GC-MS Metabolomics Retention Time Locked (RTL) library and the NIST (National Institute of Standards and Technology) mass spectra library (Ver. 2014) was performed with Unknown Analysis tool (Ver. B.08.00. Agilent Technologies). Alignment of drift (by retention time and mass) and data filtering were performed with the Mass Profiler Professional software (Ver. B.12.1, Agilent Technologies). Assignment of the target ion and the qualifiers, entire batch pre-processing and manual inspection of the acquired data including peak area and RT integration was performed with MassHunter Quantitative Analysis (Ver. B.10.00, Agilent Technologies). The dataset was interrogated to remove system contaminants. The data were evaluated for signal drift and corrected by applying a quality control-based support vector regression algorithm (QC-SVRC) (Kuligowski et al., 2015).

The metrics of the analysis quality were performed by the application of the unsupervised Principal Component Analysis (PCA) for QC sample prediction. Shewhart control charts were used to plot acquired signals versus the sample acquisition and the performance of internal standards was evaluated to overview the analytical performance. The precision of the metabolite measurements was calculated for QCs and expressed as relative standard deviation (RSD), with a cut-off value of 20% and 30% for LC-MS and GC-MS data, respectively. The assessment of data quality is presented in Supplementary Figure S2.

### Statistical analysis

Statistical analyses for metabolomics data were performed using Matlab R2015 (Mathworks) and GraphPad Prism 7 (GraphPad Software Inc., San Diego, CA). Statistical significance was assessed by ANOVA or the Kruskal-Wallis tests according to the normality of the variable distribution, with a *post hoc* test for multiple comparisons. Differences were considered statistically significant at a value of *p* < 0.05 (*p < 0.05, **p < 0.01, ***p < 0.001). Multivariate calculations and plots were performed in SIMCA-P + 16.0 (Umetrics, Umea, Sweden). A combination of VIP-p (corr) (correlation coefficient combined with VIP, Variable Influence on the Projection) based on the OPLS-DA model was applied for specified interpretations with the threshold value for variable selection set to VIP >1.0 and p (corr) > 0.4. MetaboAnalyst tool for metabolomic data analysis, visualization, and functional interpretation was used to test associations between variables and hierarchical heat map clustering (Chong et al., 2019). For clinical data evaluation, a Student’s *t*-test was applied. Stepwise forward logistic binary multivariate regression was used to account for co-correlations among clinical variables. The significance level was previously set at 95% (*p* < 0.05).

## Results

### Clinical data

The basic characteristics of the study population are presented in Table 1. In the first-trimester GDM compared with the control group, the mean maternal age, pre-gestational BMI, pregnancy BMI and parity were higher and there was a high proportion of caesarean delivery. Additionally, the GDM-related groups (I and II) delivered earlier, but their neonates’ birth weights did not vary. Glucose levels were significantly different in both GDM-related groups with a *p*-value <0.05. In the logistic binary stepwise forward regression model, the R^2^ value of 0.12 (*p* = 0.02) refers to the overall explanatory power of the model. When pregestational BMI was included as an independent variable, other parameters such as parity (*p* = 0.19), gestational BMI (*p* = 0.45), and maternal age (*p* = 0.06) lost statistical significance, indicating that their associations with maternal diabetes were mediated by their correlation with BMI. This analysis underscores pregestational BMI as the primary risk factor for maternal diabetes in our cohort.

**TABLE 1:** The basal clinical characteristics of the cohort involved in this study.

Variable	Control C	Early-onset GDM (I)	Late-onset GDM (II)	*p*-value C vs. I	*p*-value C vs. II	*p*-value I vs. II
Maternal age (years)	33.5 ± 6.16	38.8 ± 5.37	34.9 ± 4.29	0.006	ns	0.016
Parity (number)	0.6 ± 0.88	1.35 ± 1.3	0.8 ± 0.83	0.04	ns	ns
Pre-gestational BMI (kg/m^2^)	23.9 ± 4.25	29.8 ± 6.12	25.2 ± 6.15	0.001	ns	0.025
Pregnancy BMI (kg/m^2^)	28.3 ± 3.46	33.9 ± 6.43	29 ± 5.60	0.002	ns	0.015
Weight gain (kg)	11.7 ± 4.96	10.4 ± 5.34	10.1 ± 3.85	ns	ns	ns
Fasting glucose 1st trim (mg/dL)	75.8 ± 5.63	93.9 ± 13.74	82.7 ± 9.79	<0.001	0.01	0.006
Basal glucose 2nd trim (mg/dL)	76 ± 5.15	94.3 ± 14.04	85.5 (7.32)	0.001	<0.0001	ns
1 h glucose 1st trim (GCT) (mg/dL)	110.7 ± 20	184.3 (30.6)	149.5 (16.4)	<0.0001	<0.0001	0.001
Gestational age at delivery (weeks)	39.5 ± 1.49	38.3 ± 1.72	37.5 ± 3.04	0.032	0.013	ns
Cesarean delivery, n (%)	1 ± 5.26	7 ± 35	4 ± 20	0.017	ns	ns
Birth weight (kg)	3,161.5 ± 436.07	3,106 ± 758.46	2,830.2 ± 714.35	ns	ns	ns

Presented data are mean ± SD; Results were considered significant when p < 0.05.

### Metabolomics analysis

The lipidomics analysis considered 156 lipid species belonging to the class of glycerophospholipids (68 compounds, including 26 lysoglycerophospholipids, 31 glycerophosphocholines, 11 glycerophosphoethanolamines), sphingolipids (29 compounds, including 4 ceramides and 25 sphingomyelins), cholesteryl esters (11 compounds) and glycerolipids (48 compounds, including 8 diacylglycerols and 40 triacylglycerols). GC-MS-based metabolomics analysis identified a total of 49 compounds mostly belonging to the class of organic acids, fatty acids, carbohydrates, amino acids and derivatives. Statistical analysis revealed significant metabolic profile differences associated with glucose intolerance in both in early-onset and late-onset GDM. Following univariate and multivariate statistical analysis 70 metabolites were significantly increased or decreased (*p*-value <0.05 or p (corr) > 0.4 and VIP >1.0), in the specified comparison (C *vs.* I, control group compared to early-onset GDM; C *vs.* II, control group compared to late-onset GDM; I *vs.* II, the differences between early-onset and late-onset GDM). The statistical significance and metabolic changes associated with underlying diabetes are detailed in Tables 2, 3 and Figures 3, 4. Hexose levels were significantly elevated in both early-onset (*p* < 0.001) and late-onset GDM (p < 0.01). Several lipid species exhibited marked dysregulation, with the most notable changes observed in diacylglycerols such as DG (32:0), DG (34:0), DG (34:1), DG (36:1), and DG (36:2), as well as triacylglycerols, including TG (52:5), TG (56:5), TG (60:8), and TG (60:10), which showed 1.5- to 2.4-fold increases (*p* < 0.001). In late-onset GDM, our analysis revealed a 4.0-fold increase in TG (44:0), TG (46:0), and TG (46:1) (*p* < 0.001), while TG (46:2) displayed a striking 4.7-fold increase (*p* < 0.0001). Additionally, significant changes were observed in glycerophospholipids, with PC (38:3) and PC (40:4) showing a 1.4-fold increase (*p* < 0.001), and PE (34:1), PE (34:2), and PE (36:2) exhibiting 1.5-fold increases (*p* < 0.001), specifically in late-onset GDM.

**TABLE 2:** The list of the metabolites found to be significant in GC-MS analysis.

Compound name	*p*-value ANOVA	*p*-value			p (corr) and VIP						Changes [%]		
		C vs. I	C vs. II	I vs. II	C vs. I		C vs. II		I vs. II		C vs. I	C vs. II	I vs. II
2-Hydroxybutyric acid	ns	ns	ns	ns	0.6	1.2	ns	ns	0.5	1.5	+26	+4	−17
3-Hydroxybutyric acid	ns	ns	ns	ns	0.5	1.6	ns	ns	0.7	2.5	+51	−10	−41
Glycerol	ns	ns	ns	ns	0.6	1.5	ns	ns	0.6	1.8	+16	+3	−11
Urea	ns	ns	ns	ns	0.6	1.6	0.7	2	ns	ns	−21	−15	+8
2-butyne-1,4-diol	ns	ns	ns	ns	0.6	1.5	0.7	1.9	ns	ns	−21	−16	+6
p-cresol	ns	ns	ns	ns	0.5	1.1	ns	ns	ns	ns	−26	−11	+22
L-Proline	ns	ns	ns	ns	0.5	1.1	0.7	1.6	ns	ns	−12	−11	+2
L-5-Oxoproline	2.20E-04	8.41E-05	2.05E-03	ns	0.6	1	ns	ns	ns	ns	−21	−16	+6
Creatinine	4.59E-02	ns	1.35E-02	ns	ns	ns	0.6	2.3	ns	ns	−29	−62	−47
Glycine	2.42E-02	1.08E-02	3.29E-02	ns	0.6	1.4	0.6	1.3	ns	ns	−26	−21	+6
Asparagine	1.14E-03	4.16E-04	6.26E-03	ns	0.5	1.4	0.6	2.1	ns	ns	−43	−33	+18
L-Tryptophan	ns	ns	ns	ns	0.5	1	0.5	1.2	ns	ns	−12	−13	−1
L-serine	2.32E-02	3.50E-02	8.00E-03	ns	ns	ns	ns	ns	ns	ns	−11	−12	−1
L-Phenylalanine	ns	4.01E-02	ns	ns	0.7	1	ns	ns	ns	ns	−12	−9	+4
2-Methylalanine	2.88E-02	ns	8.55E-03	ns	ns	ns	0.6	1.4	ns	ns	−17	−28	−13
N-Methylguanine	2.46E-02	2.46E-02	ns	ns	0.5	1.8	ns	ns	ns	ns	−71	−44	+97
Palmitoleic acid	ns	ns	ns	ns	0.7	2.1	ns	ns	0.7	2.8	+11	−3	−12
Palmitic acid	ns	ns	ns	ns	0.7	1.5	ns	ns	0.8	2.1	+19	+2	−15
Linoleic acid	ns	ns	ns	ns	0.7	1.8	ns	ns	0.7	2.2	+26	−6	−25
Oleic acid	ns	ns	ns	ns	0.7	2	ns	ns	0.8	2.9	+26	−3	−23
Stearic acid	ns	ns	ns	ns	0.6	1	ns	ns	0.6	1.2	+10	+6	−4
Hexose	2.49E-03	8.35E-04	1.23E-02	ns	0.5	1.2	0.5	1.2	ns	ns	+43	+31	−8

Percentage of the changes in the specified comparison. The sign indicates the direction of change in the diabetes-associated groups: group I, early-onset GDM or group II, late-onset GDM.

**TABLE 3:** The list of the metabolites found to be significant in LC-MS/MS analysis.

Compound name	*p*-value ANOVA	*p*-value			p (corr) and VIP						Changes [%]		
		C vs. I	C vs. II	I vs. II	C vs. I		C vs. II		I vs. II		C vs. I	C vs. II	I vs. II
PC (34:2e)	ns	ns	ns	ns	0.6	1.2	0.5	1.0	ns	ns	−22	−16	+7
PC (38:3)	9.90E-03	ns	7.80E-03	ns	ns	ns	0.5	1.2	ns	ns	+23	+39	+13
PC (40:4)	3.11E-02	ns	2.61E-02	ns	ns	ns	0.5	1.0	ns	ns	+16	+32	+14
PC (40:7)	3.35E-02	2.85E-02	ns	ns	0.5	1.1	ns	ns	0.4	1.0	−19	−9	+13
PC(40:8)	2.60E-02	2.34E-02	ns	ns	0.5	1.0	ns	ns	ns	ns	−18	−12	+8
PE (34:1)	1.41E-02	ns	1.13E-02	ns	ns	ns	0.6	1.2	0.5	1.7	+20	+46	+22
PE (34:2)	3.50E-02	ns	2.98E-02	ns	ns	ns	0.5	1.2	0.4	1.8	+24	+46	+18
PE (36:2)	3.68E-02	ns	3.19E-02	ns	ns	ns	0.5	1.2	0.4	1.5	+20	+37	+14
PE (36:3)	3.47E-02	ns	4.15E-02	4.15E-02	ns	ns	ns	ns	0.6	2.2	+1	+29	+27
PE (36:4)	8.20E-03	ns	9.40E-03	3.15E-02	ns	ns	0.6	1.2	0.4	2.2	+10	+42	+29
PE (40:6)	2.49E-02	ns	2.05E-02	ns	ns	ns	0.5	1.2	ns	ns	+24	+46	+18
SM (d18:0/18:0)	4.00E-03	2.98E-02	3.70E-03	ns	0.4	1.1	0.7	1.2	ns	ns	+31	+45	+10
Cer (d18:1/22:0)	1.35E-02	3.96E-02	ns	1.62E-02	ns	ns	ns	ns	0.4	2.6	+30	−8	−29
CE (16:1)	1.61E-02	3.73E-02	2.15E-02	ns	ns	ns	0.6	1.2	ns	ns	+40	+48	+6
CE (18:3)	2.42E-02	ns	2.09E-02	ns	0.4	1.1	0.6	1.2	ns	ns	+30	+43	+10
CE (16:2)	9.50E-03	ns	7.70E-03	ns	ns	ns	0.6	1.4	0.5	1.3	+34	+56	+16
DG (36:2)	1.80E-03	3.20E-03	3.20E-03	ns	0.7	1.7	0.7	1.4	ns	ns	+59	+60	+1
DG (32:0)	1.80E-03	6.70E-03	3.20E-03	ns	0.6	2.0	0.7	1.8	ns	ns	+112	+144	+15
DG (34:0)	2.00E-03	5.80E-03	4.20E-03	ns	0.6	1.7	0.7	1.6	ns	ns	+76	+91	+8
DG (34:1)	9.00E-04	2.70E-03	1.90E-03	ns	0.7	2.1	0.8	1.8	ns	ns	+97	+107	+5
DG (36:1)	4.00E-04	8.00E-04	8.00E-04	ns	0.7	1.9	0.7	1.6	ns	ns	+76	+74	−1
DG (36:3)	2.08E-02	2.74E-02	4.78E-02	ns	0.7	1.7	0.4	1.1	ns	ns	+50	+45	−3
DG (38:5)	2.50E-03	5.30E-03	5.30E-03	ns	0.8	2.0	0.6	1.5	ns	ns	+73	+78	+3
TG (44:0)	2.03E-02	ns	1.59E-02	ns	ns	ns	0.6	2.3	0.5	3.0	+109	+295	+89
TG (46:0)	1.19E-02	ns	1.01E-02	ns	ns	ns	0.6	2.3	ns	ns	+154	+298	+57
TG (46:1)	2.16E-02	ns	1.78E-02	ns	ns	ns	0.6	2.1	ns	ns	+189	+291	+35
TG (46:2)	4.80E-03	ns	3.40E-03	ns	ns	ns	0.7	2.4	ns	ns	+188	+371	+64
TG (48:0)	7.90E-03	3.14E-02	9.20E-03	ns	ns	ns	0.6	2.0	ns	ns	+120	+168	+22
TG (48:1)	3.30E-03	3.22E-02	2.90E-03	ns	0.4	1.9	0.7	2.2	ns	ns	+134	+214	+35
TG (48:2)	4.70E-03	5.35E-02	3.80E-03	ns	0.4	1.6	0.7	2.1	ns	ns	+111	+199	+42
TG (48:3)	4.92E-02	ns	4.35E-02	ns	ns	ns	0.6	1.7	ns	ns	+71	+140	+40
TG (50:0)	2.80E-03	1.37E-02	3.70E-03	ns	0.4	1.7	0.7	1.7	ns	ns	+82	+98	+9
TG (50:1)	1.12E-02	2.86E-02	1.50E-02	ns	0.5	1.7	0.7	1.7	ns	ns	+83	+101	+10
TG (50:2)	4.10E-03	2.35E-02	4.50E-03	ns	0.5	1.6	0.7	1.7	ns	ns	+82	+98	+9
TG (50:3)	6.50E-03	3.60E-02	6.40E-03	ns	0.6	1.5	0.6	1.6	ns	ns	+64	+88	+15
TG (51:0)	9.50E-03	6.25E-02	8.40E-03	ns	ns	ns	0.7	2.1	0.4	1.8	+79	+157	+43
TG (52:1)	7.00E-04	1.70E-03	1.70E-03	ns	0.6	1.9	0.8	1.7	ns	ns	+74	+78	+2
TG (52:2)	6.00E-03	8.60E-03	8.60E-03	ns	0.6	1.5	0.6	1.3	ns	ns	+54	+55	+1
TG (52:3)	1.30E-02	1.80E-02	1.80E-02	ns	0.6	1.3	0.5	1.0	ns	ns	+34	+33	−1
TG (52:5)	8.80E-03	1.48E-02	1.48E-02	ns	0.7	1.8	0.5	1.5	ns	ns	+46	+96	+34
TG (52:6)	8.90E-03	ns	6.80E-03	ns	0.5	1.6	0.6	2.0	ns	ns	+93	+154	+31
TG (54:1)	5.00E-03	1.40E-02	8.20E-03	ns	0.5	1.6	0.6	1.5	ns	ns	+64	+71	+4
TG (54:2)	1.03E-02	3.27E-02	1.25E-02	ns	0.5	1.3	0.6	1.3	ns	ns	+44	+55	+8
TG (56:4)	1.86E-02	ns	2.03E-02	ns	0.5	1.3	0.6	1.4	ns	ns	+38	+51	+9
TG (56:5)	1.10E-03	3.80E-03	2.00E-03	ns	0.7	1.8	0.7	1.5	ns	ns	+49	+56	+5
TG (56:6)	1.17E-02	1.44E-02	1.44E-02	ns	0.8	1.4	0.5	1.0	ns	ns	+32	+32	0
TG (60:10)	1.88E-02	2.40E-02	4.68E-02	ns	0.7	1.9	0.4	1.2	ns	ns	+79	+60	−11
TG (60:8)	1.80E-03	3.30E-03	3.30E-03	ns	0.8	2.5	0.6	1.9	ns	ns	+125	+142	+7

Percentage of the changes in the specified comparison. The sign indicates the direction of change in the diabetes-associated groups: group I, early-onset GDM or group II, late-onset GDM.

**FIGURE 2:**
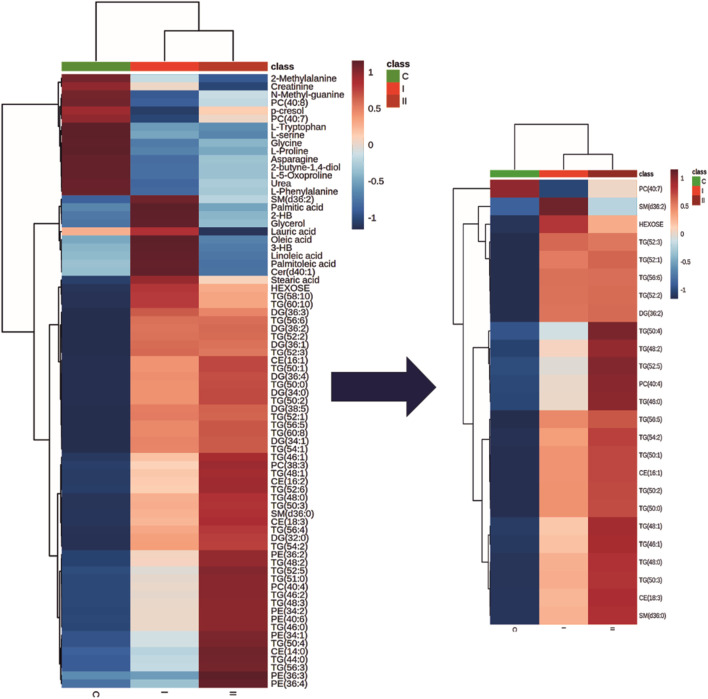
The heatmap and dendrogram show the metabolic differences and a clear cluster formation between control (C) and groups associated with diabetes (I and II). Only metabolites which were significantly associated with diabetes are presented. Each coloured cell on the map corresponds to an average of the relative metabolite abundance in the specified group (blue, the lowest; red the highest). The top 25 discriminative metabolites based on the PLS-DA VIP projection are highlighted. Rows: metabolites; Columns: experimental groups (C, green; I, bright red, II dark red). Hierarchical clustering based on Euclidean distances and Ward clustering algorithm.

**FIGURE 3:**
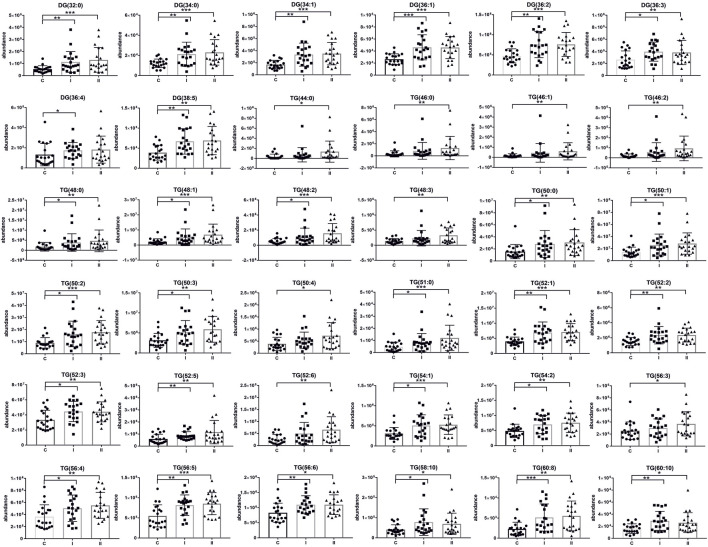
Scatter plots of the relative abundances for the group of glycerolipids representing observed changes between control (C) and diabetic groups (I, early-onset GDM and II, late-onset GDM). mean ± SD; *P*-values *p < 0.05, **p < 0.01, ***p < 0.001.

**FIGURE 4:**
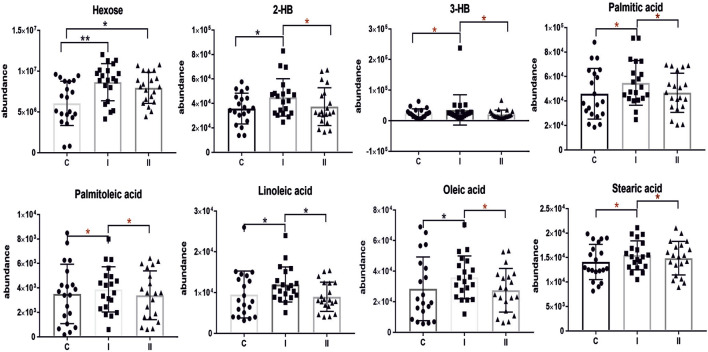
Scatter plots of the relative abundances for selected metabolites representing observed changes between control (C) and diabetic groups (I, early-onset GDM and II, late-onset GDM). The red asterisk indicates the significance according to multivariate analysis. mean ± SD; *P*-values *p < 0.05, **p < 0.01, ***p < 0.001.

**FIGURE 5:**
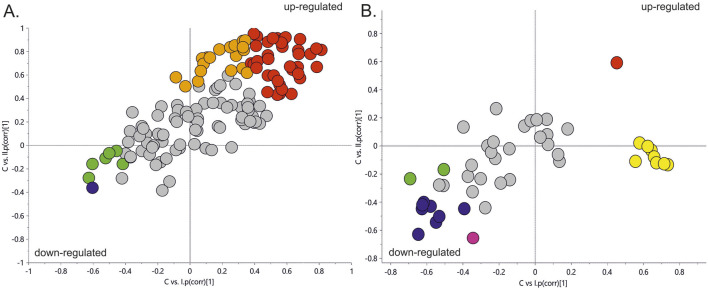
The SUS plot is based on two OPLS models (C *vs.* I and C *vs.* II). The coordinates of each variable are their correlation coefficients to the predictive components derived from each model. The position of the variables on the plot reflects their relationships to the responses of specified models. The X-axis is the predictive component of early-onset GDM, and the Y-axis is the predictive component associated with late-onset GDM. Panel **(A)**. SUS plot for LC-MS/MS data. Panel **(B)**. SUS plot for GC-MS data. Red dots–metabolites upregulated in both groups (I and II); Orange dots, represent metabolites upregulated, specific for group II; Yellow dots, metabolites upregulated, specific for group I; Navy blue dots, metabolites downregulated in both groups (I and II); fuchsia colour dot, downregulated in group II; green dots, metabolites downregulated in early-onset GDM women (I).

Figure 2 represents a specific metabolic signature associated with glucose disturbance during pregnancy. The constructed heatmap revealed considerable differences between healthy pregnant women (C) and those with early-onset GDM (I), what is even more important these differences could be noticed between control and late-onset GDM (II). It could be seen the diabetes-affected groups (I and II) are clustered together, and the metabolite levels significantly differ from the control (C). Additionally, the PLS-DA VIP projection algorithm ranked 25 metabolites to retain the most contrasting metabolic patterns (Figure 2). Among these, several glycerolipids species, including diacylglycerols (DG) and triacylglycerols (TG), belong to the group of metabolites particularly associated with observed pregnancy glucose disturbances. As depicted in Figure 3 and detailed in Table 3 all reported DG and TG were highly elevated, in both GDM-associated groups (I and II). Moreover, in most cases, those changes were also statistically significant, indicating their possible predictive proprieties. The glucose levels measured in GC-MS were statistically elevated in both GDM-related groups (Figure 4). Other compounds like organic hydroxy acids and fatty acids, with prominent changes under impaired glucose metabolism, are depicted in Figure 4. Interestingly, the Shared and Unique Structures (SUS) plot presented in Figure 5, based on two OPLS models (C *vs.* I and C *vs.* II), further emphasizes the shared and distinct metabolic alterations, capturing consistent relationships among variables. Metabolites consistently upregulated in both groups (I and II) included diacylglycerols (e.g., DG (32:0), DG (34:0), DG (34:1), DG (36:1), DG (36:2), DG (36:3), DG (36:4), DG (38:5)), triacylglycerols (e.g., TG (48:1), TG (48:2), TG (50:0), TG (50:1), TG (50:2), TG (50:3), TG (50:4), TG (52:1), TG (52:2), TG (52:3), TG (52:4), TG (52:5), TG (52:6), TG (54:1), TG (54:2), TG (56:4), TG (56:5), TG (56:6), TG (56:7), TG (56:8), TG (58:10), TG (60:8), TG (60:10)), as well as phosphatidylcholines (PC(36:4), PC(38:4)), phosphatidylethanolamines (PE (38:4), PE (40:6)), sphingomyelins (SM (d18:0/18:0)), cholesteryl ester (CE (18:3)), and hexose. These metabolites are visually represented as red dots. Conversely, metabolites consistently downregulated in those both groups, represented as navy blue dots, included L-alanine, glycine, 2-methylalanine, urea, 2-butyne-1-4-diol, L-5-oxoproline, asparagine, N-methylguanine, and PC(34:2e). Elevated levels of saturated and unsaturated fatty acids, including palmitic acid, palmitoleic acid, linoleic acid, oleic acid, and stearic acid, along with hydroxy acids such as 2-hydroxybutyric acid, 3-hydroxybutyric acid, and glycerol (yellow), combined with decreased levels of PC (34:1e), PC (36:2), PC (36:2e), PC (40:7), PC (40:8), SM (d18:1/21:0), phenylalanine, and p-cresol (green), are distinctive features of early-onset GDM. Metabolites uniquely upregulated in group II (late-onset GDM), represented by orange dots, include PC (30:0), PC (38:3), PC (40:4), PE (34:1), PE (34:2), PE (36:2), PE (36:3), PE (36:4), PE (38:6), CE (16:1), CE (14:0), CE (16:2), TG (44:0), TG (46:0), TG (46:1), TG (46:2), TG (48:0), TG (48:3), and TG (51:0). In contrast, creatinine, shown as fuchsia, was downregulated and appears to be associated with late-onset GDM.

## Discussion

There is no doubt that gestational diabetes is a major obstetrical clinical problem carrying a significant health burden for both the mother and the child. Growing evidence suggests that GDM imposes a considerable risk of developing type 2 diabetes mellitus (T2DM) postpartum among other conditions (Khan et al., 2019). Therefore, early recognition of GDM opens a window for better management of affected women and their babies (Adam et al., 2023). In most cases, GDM is the result of impaired glucose tolerance due to pancreatic β-cell dysfunction and shares the pathophysiological mechanisms with metabolic disorders associated with insulin resistance as metabolic syndrome, obesity or T2DM (Plows et al., 2018). Several risk factors contribute to the development of gestational insulin resistance, including placental, hormonal, genetic, and epigenetic variables, alongside an increase in visceral adipose tissue, changes in gut microbiota, and the coexistence of overweight or obesity (Kampmann et al., 2019). Understanding the metabolic disturbances underlying GDM holds promise for advancing our knowledge of its pathophysiology and developing targeted interventions to mitigate its adverse effects on maternal and fetal health. This insight is crucial for identifying women at risk of metabolic complications, enabling tailored prevention and personalized treatment strategies.

In our study, we look into the metabolic profile of pregnant women in the first trimester of pregnancy. The value of our research design was that we were able to select a group of asymptomatic in the first trimester individuals who had GDM diagnosed in later pregnancy (late-onset GDM). Our study offers a glimpse into molecular mechanisms of disease and enables the identification of compounds that could serve as novel players for early GDM prediction.

Advanced maternal age is recognized as a contributing factor to the development of gestational diabetes mellitus. Our study population consists of controls and women who were diagnosed with GDM in the first trimester (early onset) or a group diagnosed with GDM in the second trimester (late onset). Since we initially cross-matched the control group and the 2nd-trimester GDM group for maternal age, a potential association between maternal age and risk for GDM could only be looked for in the comparison between first-trimester GDM group and controls on one hand and 1st-trimester GDM and 2nd-trimester GDM on the other. We found statistical differences in both aforementioned comparisons that confirm the increased risk for GDM with maternal age. This finding is in line with a comprehensive systemic review and meta-analysis presented by Li et al. (2020) which involves the data on 127,275,067 participants and demonstrates a clear linear association between maternal age and the risk for GDM. In our study group, the parity was also a significantly different variable when controls were compared to 1st-trimester GDM. The control group had a lower parity than the 1st-trimester GDM group. There were no statistically significant differences between controls and 2nd-trimester GDM or between 1st- and 2nd-trimester GDM. The literature review shows little or no impact of parity on subsequent non-insulin dependent diabetes mellitus (NIDDM) which would be the case of the women with newly diagnosed or 1st-trimester GDM in our group. It is known that most women with sufficient pancreatic β-cell population tolerate well pregnancy-related insulin resistance. However, according to Peters et al., previous GDM increases close to threefold the risk of NIDDM (Peters et al., 1996) and our data on the history of previous GDM in parous women is based on self-reported information which could have a certain bias. The differences in parity appear to align, to some extent, with variations in maternal age and pre-gestational BMI, both of which are well-established risk factors for GDM. A high pre-gestational BMI not only elevates the risk of hypertensive disorders during pregnancy but also contributes to GDM by exacerbating physiological pregnancy-induced insulin resistance. As noted earlier, the control and second-trimester GDM groups were matched for pre-gestational BMI. Thus, comparisons between the first-trimester GDM group and the controls, as well as between the first- and second-trimester GDM groups, revealed statistically significant differences. Interestingly the median birth weight in our study was not elevated and differences in the newborn birth weight are neither significant between controls and GDM-related groups (I and II). Therefore we could not associate high triglyceride levels observed in our study, with increased newborn birth weight, macrosomia or large for gestational age as was concluded by other authors (Vrijkotte et al., 2011; Whyte et al., 2013; Zhu et al., 2022). This could be associated with the sample size and proper pregnancy management. Maternal lipid metabolism undergoes significant adaptations during pregnancy to meet the increased energy demands of the developing fetus and to ensure proper fetal development (Soma-Pillay et al., 2016). There is no doubt that we should still deepen our understanding of the complex interplay between lipid metabolism and other pathophysiological mechanisms, such as insulin resistance or placental dysfunction. The rise in triglyceride levels is a result of increased synthesis by the liver and reduced enzymatic activity of lipoprotein lipase, leading to decreased catabolism of adipose tissue (Soma-Pillay et al., 2016). Increased triglycerides were correlated with impaired glucose metabolism in muscle tissue and inhibited insulin signalling pathways, leading to insulin resistance (Yaribeygi et al., 2019). Insulin promotes TG storage by driving the differentiation of pre-adipocytes into mature adipocytes enhancing lipogenesis through ADD-1/SREBP-1c which regulates genes for fatty acid synthesis and lipogenesis in adipocytes and the liver facilitating glucose transport for conversion into triglycerides and inhibiting lipolysis to prevent triglyceride breakdown (Kahn and Flier, 2000; Yaribeygi et al., 2019). Our findings in alterations in TG levels in the early stages of GDM-affected pregnancies are in line with a vast literature that reported the association between high TG during pregnancy and increased risk of GDM (Li et al., 2014; Furse et al., 2019; Zhu et al., 2020). Hou et al. in a case-control study of 100 GDM and 100 normal glucose tolerance women defined the lipidomic signature in plasma across pregnancy, and proposed new lipid biomarkers for GDM prediction. The authors conclude that particularly diacylglycerols and triacylglycerols were upregulated across three trimesters of pregnancy, and demonstrated good performance in the prediction of GDM in the first and the second trimesters (Hou et al., 2023). Our data support those findings, making this evidence stronger. Moreover, Hu et al. in a large meta-analysis investigated the outcome of 292 studies, comprising 97,880 pregnant women (28,232 GDM and 69,648 controls) and also concluded that women with GDM had significantly higher TG levels that occurred in the first trimester and persisted afterwards (Hu et al., 2021).

Several studies indicate that high levels of sphingolipids, including ceramides and sphingosine-1-phosphate are correlated with pregnancy complications including gestational diabetes (Fakhr et al., 2021; Enthoven et al., 2023; Hou et al., 2023). Those bioactive compounds have been implicated in the regulation of insulin signalling pathways. Ceramides represent a major subclass of sphingolipids that interfere with insulin signalling by inhibiting Akt phosphorylation and promoting serine phosphorylation of insulin receptor substrate-1 (IRS-1) (Kanety et al., 1996). Ceramide triggers β-cell apoptosis by enhancing the permeability of the mitochondrial membrane, leading to the activation of the intrinsic apoptosis pathway that, significantly contributes to the pathogenesis of diabetes (Galadari et al., 2013; Hammad and Lopes-Virella, 2023). The results of our study are in line with the literature and indicate elevated levels of Cer(d18:1/22:0) in the group of late-onset GDM, whereas the level of SM(d18:0/18:0) increased in both GDM-related groups.

We also found the relationship between plasma levels of glycerophospholipids such as phosphatidycholines and phosphatidylethanolamines and GDM. Dysregulated glycerophospholipid metabolism has been linked to inflammation which is recognized as a key feature of diabetes. Many of those compounds can serve as precursors for pro-inflammatory lipid mediators that can activate inflammatory signalling pathways and promote the production of inflammatory cytokines, contributing to insulin resistance. According to the literature disrupted glycerophospholipid metabolism is common in GDM (Dudzik et al., 2014; Liu et al., 2016; Zhan et al., 2021), however, due to the diversity of glycerophospholipids structures further research is needed to elucidate the specific role in GDM molecular mechanisms.

Our data confirm the significance of fatty acids in the pathophysiology of GDM which is consistent with our first data from the study performed in GDM plasma from the second trimester of pregnancy (Dudzik et al., 2014; Dudzik et al., 2017) and others (Chen et al., 2010; Scholtens et al., 2014; Enquobahrie et al., 2015). All reported fatty acids including saturated palmitic acid (C16:0), stearic acid (C18:0) and unsaturated linoleic acid (ω-6, C18:2), palmitoleic acid (ω-7, C16:1), oleic acid (ω-9, C18:1) were elevated in the group of early-onset GDM, however, this trend was not observed in late-onset GDM. Several studies confirm that the circulation of maternal free fatty acids (FFAs) plays an important role in the pathophysiology of GDM due to their involvement in various metabolic processes. Elevated levels of FFAs in pregnancy inhibit total body glucose uptake and oxidation. Chronic exposure to high levels of FFAs can impair pancreatic β-cell function leading to reduced insulin secretion and insulin resistance (Sivan et al., 1998; Sun et al., 2022). Meta-analysis and original data presented by Sun et al. indicate that GDM women are characterized by a particular circulating saturated FA profile with altered levels of palmitic acid and lower levels of very-long-chain FA. The results demonstrated that palmitic acid has a strong positive correlation with GDM both in the early and second trimesters of pregnancy (Sun et al., 2022). Other studies conducted by Ogundipe et al. have shown that GDM has a unique fatty acid profile with elevated levels of omega 6 fatty acids compared to omega 3 an abnormal pattern of sequential *n*-6 metabolism (Ogundipe et al., 2020).

Special attention should be also placed on hydroxy acids, namely, 2-hydroxybutyric acid (2-HB) and 3-hydroxybutyric acid (3-HB). Its specific role in the pathological process that leads to GDM has not been extensively studied so far however, there are several potential mechanisms through which those compounds may contribute to this condition. Gall et al. postulated that 2-HB, an organic acid derived from 2-ketobutyric acid, could be an early indicator for both insulin resistance (IR) and impaired glucose regulation (Gall et al., 2010). Elevated 2-HB is strongly linked to impairment of β-cells function and may reflect disruptions in metabolic pathways, such as increased fatty acid oxidation, ketogenesis and oxidative stress which are common features of insulin resistance state and GDM (Gall et al., 2010; Sousa et al., 2021). Moreover, 2-HB has been implicated in chronic low-grade inflammation which is a hallmark feature of obesity and a major risk factor for GDM (Sousa et al., 2021). The role of the ketone body as 3-hydroxybutyric acid in the pathophysiology of GDM remains an area of active investigation. High 3-HB levels observed in GDM may reflect metabolic dysregulation including increased lipolysis and ketogenesis, which contribute to alterations in energy metabolism and glucose homeostasis. 3-HB is produced during fatty acid oxidation and serves as an alternative energy substrate (Qi et al., 2022). This compound was found to be associated with GDM reported in many metabolomics studies (White et al., 2017; Lu et al., 2021; McMichael et al., 2021; Sikorski et al., 2022). What is more interesting, hydroxybutyric acid has been connected with gut microbiota-derived metabolites showing significantly higher levels in women with GDM (Singh et al., 2023; Ye et al., 2023). Growing evidence suggests that ketone bodies may serve as immunomodulators to attenuate pathological inflammation (Qi et al., 2022). A very recent study by Neudorf et al. postulates that it is plausible that 3-HB could mitigate inflammatory signalling pathways implicated in diabetes (Neudorf et al., 2024). Considering the potential of β-HB and the intriguing literature data we believe this compound is worthy of particular attention. In our study, we observed that higher levels of both 2-HB and 3-HB were associated with early-onset GDM, although not significant in late-onset GDM. The results are consistent with our first data where we identified a panel of plasma metabolites implicated in GDM pathophysiology (Dudzik et al., 2014; Dudzik et al., 2017; Burzynska-Pedziwiatr et al., 2023), however, those studies were performed on the plasma samples from the second trimester, different cohort and diagnosis of GDM were based on different criteria. Nevertheless, in both cases, the observed changes were associated with glucose impairment and diagnosed GDM.

It is worth mentioning that our study identified other metabolites like p-cresol, 2-butyne-1,4-diol or tryptophan (Trp) linked to the intestinal microbiota. There is limited evidence linking those compounds to GDM, but their possible association with inflammation suggests that they may play a role in the molecular background of GDM. The essential amino acid L-tryptophan is particularly important in pregnancy due to the high demand for maternal protein synthesis and fetal growth and development (Badawy, 2015). Our findings from present and previous experiments show a reduced level of tryptophan in GDM (Dudzik et al., 2014), which is in line with other studies (Leitner et al., 2017; Özdemir et al., 2023). This decrease may be attributed to increased degradation or altered utilization of Trp in GDM. The recent systematic review by van Zundert et al. indicates that decreased Trp levels in maternal blood in the second and third trimester of pregnancy was associated with several pregnancy complications including gestational diabetes (van Zundert et al., 2022).

We found that changes observed in amino acid profile are not entirely consistent across studies, most likely due to differences in the trimesters of pregnancy at which the study was performed and the criteria for GDM definition. In our study, glycine, serine and proline levels were lower in the GDM group which is consistent with findings reported in previous studies performed in the first and the second trimester of pregnancy (Zhao et al., 2019; Lu et al., 2021). Nevertheless, other studies report that first-trimester, early-onset GDM was associated with higher concentrations of glycine and proline compared to the control group (Razo-Azamar et al., 2023).

Our study point also an association between significant depletion in creatinine levels and late-onset GDM. The results are concordant with the other findings (Chen et al., 2023) and literature describing a positive correlation between lower serum creatinine and abnormal glucose metabolism (Harita et al., 2009; Bao et al., 2018). Finally, our study confirmed high glucose levels observed both in the early- and late-onset GDM group.

## Conclusion

The metabolomics approach provides a powerful tool for understanding the metabolic changes associated with gestational diabetes mellitus (GDM). It offers a snapshot of the phenotypic state at the time of sampling, allowing for hypothesis generation and translational insights that can serve both researchers and clinicians in better understanding disease mechanisms and enabling earlier recognition of impaired glucose tolerance during pregnancy. Our study indicates several molecules providing biomarker candidates, especially for late-onset GDM prediction. Although several studies have been performed so far, no metabolite-based prediction factor for late-onset GDM exists, therefore, more effort is required to elucidate the molecular landscape of GDM. Nevertheless, we recognise some limitations in our study. The relatively small sample size and single-centre design may limit the significance of the findings. Expanding the study to larger, multi-centre cohorts with greater ethnic and geographic diversity would enhance the robustness of the identified metabolic patterns. Future research should address the limitations of inter-individual variability resulting from genetics, diet, and environmental factors.

## Data Availability

The original contributions presented in the study are included in the article/Supplementary Material, further inquiries can be directed to the corresponding authors.

## Glossary

AcAc: Glacial Acetic Acid
ACN: Acetonitrile
ADD1: Adipocyte Determination and Differentiation-Dependent Factor 1
BSTFA: N,O bis(trimethylsilyl)trifluoroacetamide
dMRM: dynamic Multiple Reaction Monitoring Mode
EI: Electron Impact
ESI: Electrospray Ionization
EtAc: Ethyl Acetate
FA: Formic Acid
GC/Q-TOF-MS: Gas Chromatography-Quadrupole-Time of Flight Mass Spectrometry
IRS-1: Insulin Receptor Substrate-1
IR: Insulin Resistance
IS: Internal Standard
LC-MS/MS: Liquid Chromatography-Tandem Mass Spectrometry
MeOH: Methanol
MS: Mass Spectrometry
MS/MS: Tandem Mass Spectrometry
NIDDM: Non-Insulin Dependent Diabetes Mellitus
NIST: National Institute of Standards and Technology
OPLS-DA: Orthogonal Partial Least Squares Discriminant Analysis
PCA: Principal Component Analysis
PrOH: 2-propanol
QC-SVRC: Quality Control based Support Vector Regression
RSD: Relative Standard Deviation
RT: Retention Time
RTL: Retention Time Locked
SREBP: Sterol Regulatory Element Binding Protein
SUS: Shared and Unique Structures Plot Analysis
TMCS: 1% trimethylchlorosilane
VIP: Variable Influence on the Projection
