# Supplementary material for: First-trimester metabolic profiling of gestational diabetes mellitus: insights into early-onset and late-onset cases compared with healthy controls
Source: Front Mol Biosci. 2025 Jan 15;11:1452312. doi: 10.3389/fmolb.2024.1452312 (PMC11774710; doi:10.3389/fmolb.2024.1452312)
Supplement: Supplementary file 1 [file DataSheet1.pdf]

Supplementary Information

First-trimester metabolic profiling of gestational diabetes mellitus: insights into early-onset and late-onset cases compared with healthy controls

Danuta Dudzik[1*], Vangeliya Atanasova[2], Coral Barbas[3], Jose Luis Bartha[2*]

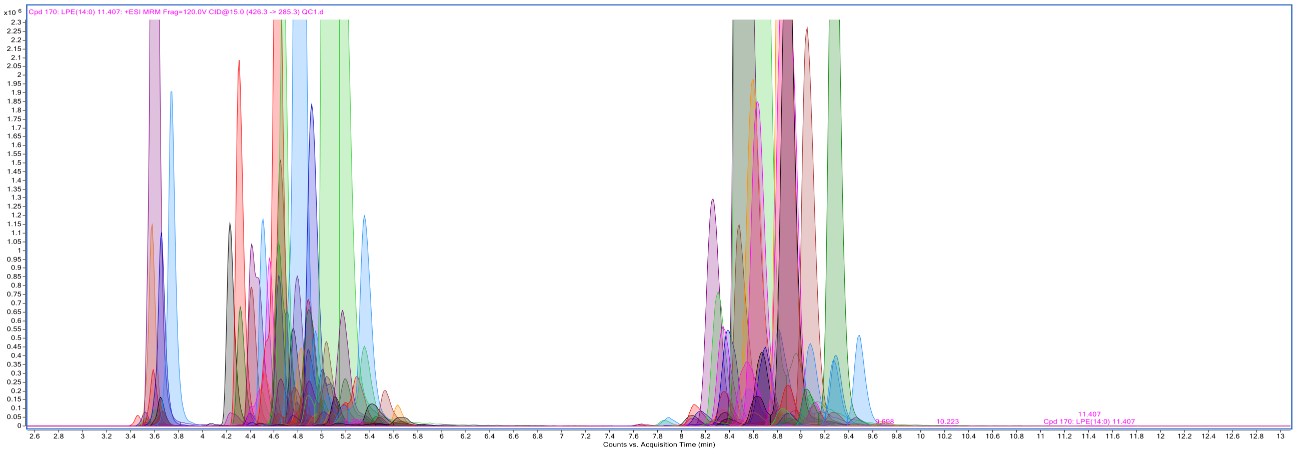

Supplementary Figure 1. Representative MRM (multiple reaction monitoring) chromatograms of targeted metabolites.

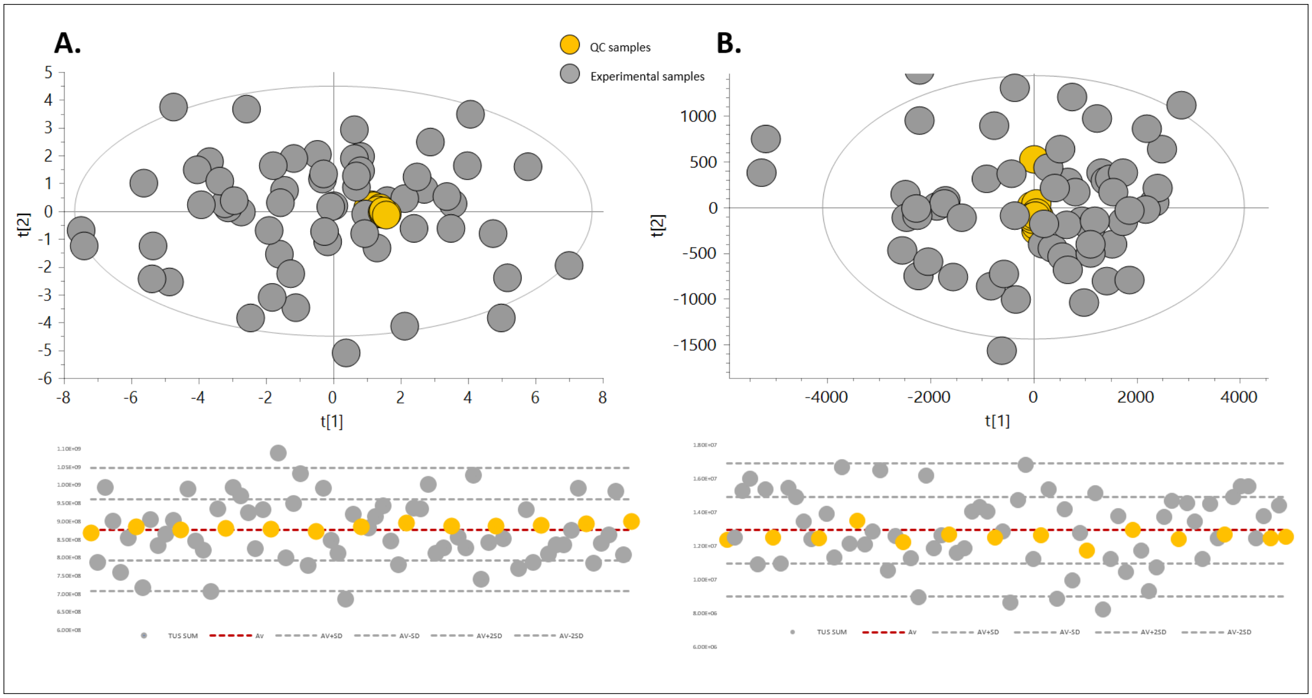

Supplementary Figure 2. Data quality assessment based on multivariate PCA-X model and evaluation of average QCs signals in the function of acquisition order. QC data points (orange dots) cluster tightly and observe mean signal for metabolites in QC samples is constant. (A) LC-MS data and (B) GC-MS data.

Supplementary Table 1. Acquisition parameters of the metabolites included in the analytical method.

Compound name	Precursor Ion	Product Ion	RT (min)	Fragmentor	Collision Energy	Cell Accelerator Voltage	Polarity
CE(16:0)	642.618	369.356	9.514	120	10	2	Positive
CE(16:1)	640.602	369.356	9.149	120	10	2	Positive
CE(18:2)	666.618	369.356	9.369	120	10	2	Positive
CE(18:3)	664.602	369.356	9.115	120	10	2	Positive
CE(20:4)	690.618	369.356	9.327	120	10	2	Positive
CE(22:5)	716.634	369.356	9.572	120	10	2	Positive
CE(22:6)	714.618	369.356	9.310	120	10	2	Positive
CE(14:0)	614.587	369.356	8.972	120	10	2	Positive
CE(15:0)	628.602	369.356	9.200	120	10	2	Positive
CE(16:2)	638.587	369.356	8.929	120	10	2	Positive
CE18:1	668.633	369.356	9.708	120	10	2	Positive
Cer(d18:0/24:0)	634.645	266.25	6.576	120	40	5	Positive
Cer(d18:1/22:0)	604.598	264.25	6.032	120	40	5	Positive
Cer(d18:1/24:0)	632.598	264.25	6.403	120	40	5	Positive
Cer(d18:1/24:1)	630.614	264.25	6.161	120	40	5	Positive
DG(18:1/18:1)	638.571	339.289	6.221	120	20	2	Positive
DG(32:0)	586.54	313.28	5.894	120	20	2	Positive
DG(34:0)	614.571	341.305	6.282	120	20	2	Positive
DG(34:1)	612.556	313.273	6.058	120	20	2	Positive
DG(36:1)	640.586	341.304	6.429	120	20	2	Positive
DG(36:3)	636.556	337.273	6.023	120	20	2	Positive
DG(36:4)	634.54	337.273	5.825	120	20	2	Positive
DG(38:5)	660.556	339.289	6.066	120	20	2	Positive
LPC(14:0)sn-1	468.308	184.076	3.488	120	30	4	Positive
LPC(15:0)	482.324	184.076	3.557	120	30	4	Positive
LPC(16:0)sn-1	496.34	184.076	3.642	120	30	4	Positive
LPC(16:0e)	482.36	184.076	3.557	120	30	4	Positive
LPC(16:1)sn-1	494.324	184.076	3.548	120	30	4	Positive
LPC(16:1e)	480.345	184.076	3.686	120	30	4	Positive
LPC(17:0)	510.355	184.076	3.702	120	30	4	Positive
LPC(17:1)	508.34	184.076	3.608	120	30	4	Positive
LPC(18:0)sn-1	524.371	184.076	3.788	120	30	4	Positive
LPC(18:0e)	510.39	184.076	3.702	120	30	4	Positive
LPC(18:0p)	508.376	184.076	3.608	120	30	4	Positive
LPC(18:1)sn-1	522.355	184.076	3.694	120	30	4	Positive
LPC(18:1e)	508.376	184.076	3.608	120	30	4	Positive

LPC(18:2)sn-1	520.34	184.076	3.607	120	30	4	Positive
LPC(18:2e)	506.361	184.076	3.548	120	30	4	Positive
LPC(18:3)	518.324	184.076	3.547	120	30	4	Positive
LPC(20:3)	546.356	184.076	3.685	120	30	4	Positive
LPC(20:4)sn-1	544.34	184.076	3.624	120	30	4	Positive
LPC(20:5)	542.33	184.076	3.556	120	30	4	Positive
LPC(22:5)	570.356	184.076	3.693	120	30	4	Positive
LPC(22:6)sn-1	568.34	184.076	3.641	120	30	4	Positive
LPE(14:0)	426.27	285.27	7.000	120	15	5	Positive
LPE(16:0)sn-1	454.293	313.274	3.626	120	15	2	Positive
LPE(18:1)sn-1	480.308	339.29	3.686	120	15	2	Positive
LPE(18:2)sn-1	478.293	337.274	3.600	120	15	2	Positive
LPE(20:4)sn-1	502.293	361.275	3.616	120	15	2	Positive
PC(30:0)	706.538	184.076	4.863	120	35	2	Positive
PC(32:0)	734.569	184.076	5.231	120	35	2	Positive
PC(32:1)	732.554	494.33	5.025	120	35	2	Positive
PC(34:0)	762.601	184.076	5.417	120	35	2	Positive
PC(34:1)	760.585	496.34	5.409	120	35	2	Positive
PC(34:1e)	746.606	184.076	5.564	120	35	2	Positive
PC(34:2)	758.569	496.34	5.187	120	35	2	Positive
PC(34:2e)	744.59	184.076	5.005	120	35	2	Positive
PC(34:3)	756.554	494.33	4.981	120	35	2	Positive
PC(36:0)	792.574	608.494	7.000	120	35	2	Positive
PC(36:0)	790.632	524.5	7.000	120	35	2	Positive
PC(36:1)	788.616	524.38	5.794	120	35	2	Positive
PC(36:2)	786.601	522.36	5.562	120	35	2	Positive
PC(36:2e)	772.62	184.076	5.340	120	35	2	Positive
PC(36:3)	785.588	185.081	5.357	120	35	2	Positive
PC(36:3e)	770.606	502.5	5.752	120	35	2	Positive
PC(36:4)	783.572	184.076	5.280	120	35	2	Positive
PC(36:4)sec	769.59	185.081	5.460	120	35	2	Positive
PC(36:5)	780.564	184.076	5.071	120	35	2	Positive
PC(38:2)	814.632	548.375	7.000	120	35	2	Positive
PC(38:3)	812.616	546.36	5.793	120	35	2	Positive
PC(38:4)	810.604	524.5	5.655	120	35	2	Positive
PC(38:4e)	796.625	528.352	7.000	120	35	2	Positive
PC(38:5)	808.588	522.362	5.467	120	35	2	Positive
PC(38:5e)	794.609	526.5	5.785	120	35	2	Positive
PC(38:6)	807.573	185.081	5.287	120	35	2	Positive
PC(40:0)	847.698	185.079	6.051	120	35	2	Positive
PC(40:4)	838.635	184.076	5.947	120	35	2	Positive
PC(40:6)	835.604	185.081	5.680	120	35	2	Positive

PC(40:7)	832.585	184.076	5.457	120	35	2	Positive
PC(40:8)	830.569	184.076	6.276	120	35	2	Positive
PE(34:1)	718.538	577.52	5.351	120	25	3	Positive
PE(34:2)	716.522	575.504	5.137	120	25	3	Positive
PE(36:2)	744.554	603.535	5.539	120	25	3	Positive
PE(36:3)	742.538	601.52	5.324	120	25	3	Positive
PE(36:3e)	728.559	392.35	5.702	120	25	3	Positive
PE(36:4)	740.522	599.504	5.205	120	25	3	Positive
PE(38:4)	768.554	627.535	5.606	120	25	3	Positive
PE(38:5e)	752.559	392.35	5.745	120	25	3	Positive
PE(38:6)	764.522	623.504	5.263	120	25	3	Positive
PE(40:6)	792.554	651.535	5.622	120	25	3	Positive
PE(40:7e)	776.559	392.35	5.778	120	25	3	Positive
SM(d16:1/17:0)	689.56	184.076	4.510	120	30	4	Positive
SM(d18:0/14:0)	677.56	184.076	4.389	120	30	4	Positive
SM(d18:0/16:0)	705.591	184.076	4.760	120	30	4	Positive
SM(d18:0/18:0)	733.653	184.076	5.017	120	30	4	Positive
SM(d18:0/24:0)	817.716	184.076	6.154	120	30	4	Positive
SM(d18:1/14:0)	675.544	184.076	4.389	120	30	4	Positive
SM(d18:1/16:0)	704.578	185.081	4.665	120	30	4	Positive
SM(d18:1/17:0)	717.591	184.076	4.820	120	30	4	Positive
SM(d18:1/18:0)	731.606	184.076	4.991	120	30	4	Positive
SM(d18:1/18:2)	727.567	184.076	4.613	120	30	4	Positive
SM(d18:1/20:0)	759.637	264.25	7.000	120	30	4	Positive
SM(d18:1/21:0)	773.653	184.076	5.588	120	30	4	Positive
SM(d18:1/22:0)	787.669	184.076	5.600	120	30	4	Positive
SM(d18:1/23:0)	801.684	184.076	5.974	120	30	4	Positive
SM(d18:1/24:0)	815.7	264.25	6.156	120	30	4	Positive
SM(d18:1/25:1)	827.7	184.076	6.017	120	30	4	Positive
SM(d18:2/16:0)	701.559	184.076	4.501	120	30	4	Positive
SM(d18:2/18:0)	729.587	184.076	4.811	120	30	4	Positive
SM(d18:2/20:0)	757.622	184.076	4.999	120	30	4	Positive
SM(d18:2/22:0)	785.653	262.25	5.536	120	30	4	Positive
SM(d18:2/23:0)	799.669	184.076	5.761	120	30	4	Positive
SM(d18:2/24:0)	814.687	185.081	5.888	120	30	4	Positive
SM(d18:2/24:1)	811.669	262.25	5.707	120	30	4	Positive
SM(d18:2/24:2)	809.653	262.25	5.467	120	30	4	Positive
SM(d19:0/24:1)	829.716	184.076	6.267	120	30	4	Positive
TG(44:0)	768.705	523.47	8.614	100	35	4	Positive
TG(46:0)	796.738	523.472	9.071	100	35	4	Positive
TG(46:1)	794.721	521.46	8.834	100	35	4	Positive
TG(46:2)	792.705	521.454	8.571	100	35	4	Positive

TG(48:0)	824.77	551.503	9.511	100	35	4	Positive
TG(48:1)	822.754	549.487	9.265	100	35	4	Positive
TG(48:2)	820.738	575.503	9.037	100	35	4	Positive
TG(48:3)	818.723	521.5	8.799	100	35	4	Positive
TG(50:0)	852.801	579.534	9.714	100	35	4	Positive
TG(50:1)	850.785	577.49	9.721	100	35	4	Positive
TG(50:2)	848.77	575.503	9.468	100	35	4	Positive
TG(50:3)	846.754	549.487	9.222	100	35	4	Positive
TG(50:4)	844.738	547.738	8.993	100	35	4	Positive
TG(51:0)	867.819	580.55	10.169	100	35	4	Positive
TG(52:1)	878.817	605.55	10.181	100	35	4	Positive
TG(52:2)	876.801	575.503	9.916	100	35	4	Positive
TG(52:3)	874.785	575.503	9.662	100	35	4	Positive
TG(52:4)	872.77	575.503	9.416	100	35	4	Positive
TG(52:5)	870.754	575.503	9.213	100	35	4	Positive
TG(52:6)	868.738	547.472	9.052	100	35	4	Positive
TG(54:1)	906.848	605.55	10.679	100	35	4	Positive
TG(54:2)	904.832	605.55	10.403	100	35	4	Positive
TG(54:3)	902.817	603.354	10.143	100	35	4	Positive
TG(54:4)	900.801	601.519	9.856	100	35	4	Positive
TG(54:5)	898.785	599.503	9.617	100	35	4	Positive
TG(54:6)	896.77	599.503	9.390	100	35	4	Positive
TG(54:7)	894.754	597.487	9.169	100	35	4	Positive
TG(56:3)	930.848	631.57	10.637	100	35	4	Positive
TG(56:4)	928.832	629.55	10.360	100	35	4	Positive
TG(56:5)	926.817	627.534	10.108	100	35	4	Positive
TG(56:6)	924.801	603.534	9.965	100	35	4	Positive
TG(56:7)	922.785	601.519	9.720	100	35	4	Positive
TG(56:8)	920.77	599.503	9.516	100	35	4	Positive
TG(58:10)	944.77	623.503	9.533	100	35	4	Positive
TG(58:6)	952.832	952.832	7.000	100	35	4	Positive
TG(58:8)	948.801	601.519	9.947	100	35	4	Positive
TG(58:9)	946.785	601.519	9.728	100	35	4	Positive
TG(60:10)	972.801	651.534	10.006	100	35	4	Positive
TG(60:12)	968.77	647.55	9.540	100	35	4	Positive
TG(60:8)	976.832	655.55	7.000	100	35	4	Positive
